# Chicoric Acid Effectively Mitigated Dextran Sulfate Sodium (DSS)-Induced Colitis in BALB/c Mice by Modulating the Gut Microbiota and Fecal Metabolites

**DOI:** 10.3390/ijms25020841

**Published:** 2024-01-10

**Authors:** Jiani Yang, Jie Lin, Ting Gu, Quancai Sun, Weidong Xu, Ye Peng

**Affiliations:** 1 School of Food and Biological Engineering, Jiangsu University, Zhenjiang 212013, China; yangjiani0411@163.com (J.Y.); guting1029@163.com (T.G.); 2 Faculty of Medicine, Macau University of Science and Technology, Taipa, Macao SAR, China; 3 Department of Nutrition and Integrative Physiology, Florida State University, Tallahassee, FL 32306, USA; lj23g@fsu.edu (J.L.); sqctp@hotmail.com (Q.S.); 4 School of Pharmacy, Jiangsu University, Zhenjiang 212013, China

**Keywords:** chicoric acid, colitis, DSS, gut microbiota, metabolites

## Abstract

Chicoric acid (CA) has been reported to exhibit biological activities; it remains unclear, however, whether CA could regulate colitis via modulation of the gut microbiota and metabolites. This study aimed to assess CA’s impact on dextran sulfate sodium (DSS)-induced colitis, the gut microbiota, and metabolites. Mice were induced with 2.5% DSS to develop colitis over a 7-day period. CA was administered intragastrically one week prior to DSS treatment and continued for 14 days. The microbial composition in the stool was determined using 16S rRNA sequencing, while non-targeted metabolomics was employed to analyze the metabolic profiles of each mouse group. The results show that CA effectively alleviated colitis, as evidenced by an increased colon length, lowered disease activity index (DAI) and histological scores, and decreased tumor necrosis factor-α (TNF-α) and interleukin-6 (IL-6) expression levels. CA intervention restored the structure of gut microbiota. Specifically, it decreased the abundance of Bacteroidetes and Cyanobacteria at the phylum level and *Bacteroides*, *Rosiarcus*, and unclassified *Xanthobacteraceae* at the genus level, and increased the abundance of unclassified *Lachnospiraceae* at the genus level. Metabolomic analysis revealed that CA supplementation reversed the up-regulation of asymmetric dimethylarginine, N-glycolylneuraminic acid, and N-acetylneuraminic acid, as well as the down-regulation of phloroglucinol, thiamine, 4-methyl-5-thiazoleethanol, lithocholic acid, and oxymatrine induced by DSS. Our current research provides scientific evidence for developing CA into an anti-colitis functional food ingredient. Further clinical trials are warranted to elucidate the efficacy and mechanism of CA in treating human inflammatory bowel disease (IBD).

## 1. Introduction

Inflammatory bowel disease (IBD) is a chronic inflammatory condition affecting the gastrointestinal mucosa, encompassing ulcerative colitis (UC) and Crohn’s disease (CD), Behcet’s syndrome and lymphocytic syndrome, and infectious or ischemic colitis [1,2]. The incidence and prevalence of UC and CD have shown a global increase over the past five decades, contributing to a significant health burden [3]. UC, characterized by idiopathic chronic relapsing–remitting inflammation in the colon that leads to symptoms like diarrhea and rectal bleeding, arises from a complex interplay of genetic, microbial, environmental, and other unidentified factors [4]. Standard treatments for ulcerative colitis primarily involve anti-inflammatory medications, including 5-aminosalicylates such as olsalazine, mesalazine, and balsalazide. For mild-to-moderate UC, immunosuppressants like thiopurines, 6-mercaptopurine, methotrexate, and cyclosporine are commonly prescribed [5]. In cases of moderate-to-severe colon inflammation, other drug classes, such as corticosteroids like prednisolone and anti-TNF-α antibodies, may also be utilized [6]. Surgery becomes a viable option for IBD patients in cases of refractory and fulminant disease stages [7]. Nonetheless, prolonged use of synthetic drugs can result in numerous complications and side-effects, including vomiting, nausea, headache, fatigue, liver and kidney toxicity, drug resistance, and allergic reactions [8,9].

In the gastrointestinal tract of mammals, gut microorganisms engage in intricate communication with intestinal epithelial cells, playing a pivotal role in the regulation of intestinal homeostasis [10]. Disruptions in the gut microbiota can adversely affect intestinal epithelial function and lead to heightened intestinal permeability [11]. Patients suffering from IBD frequently exhibit aberrations in the composition and activity of their gut microbiota, marked by reduced diversity, a decreased proportion of Firmicutes, and an increased proportion of Proteobacteria and Actinobacteria [11,12]. Moreover, the proliferation of pro-inflammatory microbiota, such as *Ruminococcus gnavus* and *Escherichia coli*, alongside the depletion of anti-inflammatory microbiota, including *Bacteroides*, *Lachnospiraceae*, and *Faecalibacterium prausnitzii*, has been associated with the progression of IBD [13]. Gender influences IBD through the gut flora, as the metabolism of androgens and estrogens is thought to be related to the gut microbiota [14,15]. One study reported that individuals, both men and women, with elevated levels of testosterone and estradiol exhibited more diverse bacterial communities. This suggests that sex hormones may contribute to the maintenance of gut health [16]. The majority of women encounter IBD symptoms during the premenstrual or menstrual cycle, pregnancy, and postpartum periods, as sex hormones may prolong the gastrointestinal transit time during the luteal phase [17]. Research has indicated that the dysregulation of bile acid metabolism and its psychological complications in CD patients may be linked to an overabundance of *Enterobacteriaceae* and *Lachnospiraceae*, and a diminished abundance of *Faecalibacterium prausnitzii* [18]. In a clinical trial involving 305 participants, the average number of *Lactobacillus*, *Bifidobacterium*, and *Bacteroides* increased after the consumption of probiotic yogurt, resulting in the restoration of intestinal function [19]. A recent experimental study demonstrated that a blend of four probiotic strains (including *L. acidophilus* LA1, *L. paracasei* 101/37, *B. animalis* spp. Lactis Bi1, and *B. breve* Bbr8) suppressed the production of IL-8, IL-23, and IL-1β cytokine in monocyte-derived dendritic cells obtained from UC patients [20]. The pronounced decrease in *Roseburia* in IBD may be associated with low levels of *R. intestinalis*, which is thought to maintain intestinal homeostasis and induce anti-inflammatory responses through IBD regulatory mechanisms [21]. Despite the robust connection between gut microbiota and IBD, the mechanisms of interaction remain incompletely elucidated. Metabolomics, frequently employed in clinical applications, serves to shed new light on physiological regulatory processes within complex mammalian systems, spanning disease etiology, diagnostic stratification, and potential mechanisms of therapeutic action. It is characterized by the exploration of intricate metabolic interactions between the host and its symbiotic microbiota [22]. Alterations in gut microbiota lead to modifications in bacterial metabolites, including the production of short-chain fatty acids (SCFA), tryptophan, and other small molecules within the gut [23]. Insufficient tryptophan levels can contribute to the development or worsening of IBD [24]. SCFAs, a category of fatty acid compounds with alkyl chains shorter than six carbons, are produced through the fermentation of anaerobic gut microbes abundant in dietary fiber substrates. They may modulate inflammation associated with IBD, a process crucial in the pathogenesis of IBD [25]. Recent research has demonstrated that butyrate can modify the gut microbiota in IBD patients, leading to a reduction in intestinal symptoms [26]. In vitro studies revealed that butyrate and propionate were more potent than acetate in suppressing lipopolysaccharide (LPS)-induced TNFα production by neutrophils and inhibiting TNFα-mediated nuclear factor kappa-B (NF-κB) activation in a human colon cancer cell line [27]. Within human colonic intestinal epithelial cells, butyrate enhances intestinal epithelial barrier function by inhibiting the activation of histone deacetylase, hypoxia-inducible factor-1, and signal transducer and activator of transcription-3. This modulation contributes to the integrity of epithelial tight junctions and hinders LPS-induced NF-κB activation [3]. Importantly, SCFAs can additionally impede the growth of potentially invasive pathogenic bacteria, such as *Escherichia coli*, by influencing intracellular pH and metabolic functions [28]. Consequently, it is crucial to understand the influence of intestinal microbiota and their metabolites on colitis development.

Chichoric acid (CA), also known as dicaffeoyl tartaric acid, was initially identified in 1958 [29,30]. CA is a natural phenolic compound, one of the important active ingredients in *Echinacea purpurea* and Cichorium intybus, reported to have anti-inflammatory, antioxidant, and antiviral activities [31]. CA has been demonstrated to attenuate lipopolysaccharide-induced inflammatory signaling nuclear factor kappa-B p65 (NF-κB p65) and cyclooxygenase-2 activity by inhibiting leucine-rich repeat (LRR) and pyrin domain (PYD) -containing protein 3 inflammasome activation, inhibiting caspase-1 activity, and inhibiting the inflammasome-mediated secretion of interleukin-10 and interleukin-18 [32]. In a model of LPS-induced acute lung injury, CA has been shown to reduce inflammatory cell infiltration, myeloperoxidase activity, and proinflammatory cytokine production in bronchoalveolar lavage fluid. Furthermore, in a rat model of collagen-induced arthritis, treatment with CA extracts from *Echinacea purpurea* (at doses of 8, 16, and 32 mg/kg) significantly reduced serum levels of TNF-α, interleukin-1β (IL-1β), and Prostaglandin E2 (PGE-2), leading to substantial relief in paw swelling [33]. However, the influence of CA on colitis, gut microbiota, and metabolite dysregulation induced by DSS remains to be determined.

Therefore, in this study, we aim to study the influence of CA on colitis induced by DSS in BALB/c mice. To further understand the potential role of gut microbiota and metabolites, 16S rRNA sequencing and fecal metabolomics were applied to elucidate the alterations in microbial community structure and crucial metabolites after DSS exposure as well as CA supplementation.

## 2. Results

### 2.1. Supplementation with CA Alleviated DSS-Induced Colitis in Mice

**Figure 1:**
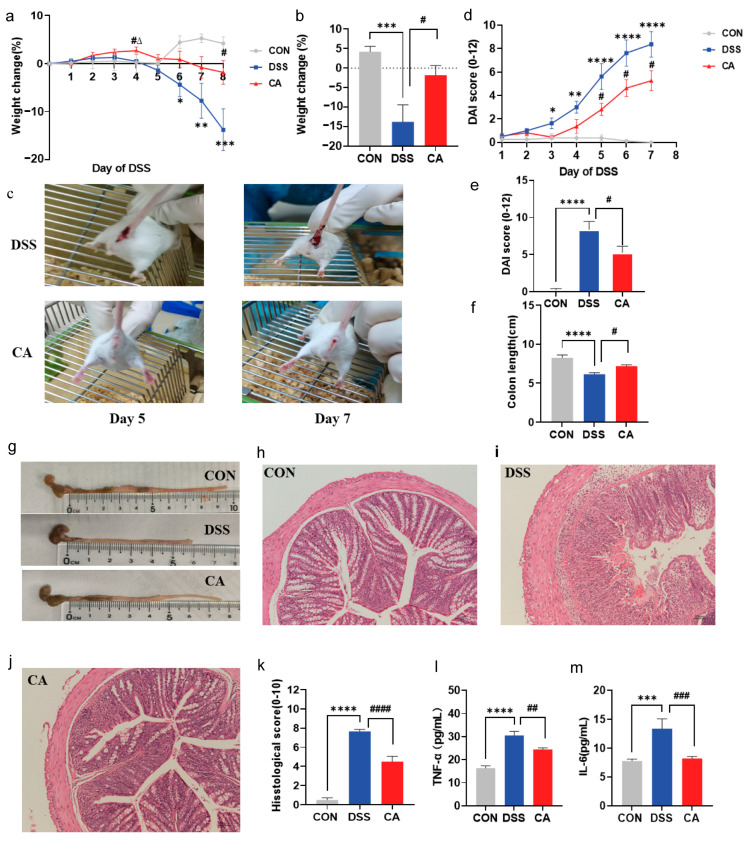
Supplementation with CA alleviated DSS-induced colitis. (**a**) Percent change in mouse body weight during DSS administration. (**b**) The percentage change in mouse body weight after seven days of DSS administration. (**c**) Presence of blood in the feces of mice on the fifth and seventh days of DSS modeling. (**d**) Disease activity index (DAI) scores of mice during DSS administration. (**e**) DAI score of mice after seven days of DSS modeling. (**f**) Colon length measurement in each group. (**g**) Representative colon from each group. (**h**–**j**) Representative images of H&E-stained colon (100× magnification). (**k**) Histological scores of colon in each group. (**l**,**m**) Expression levels of *TNF-α* and IL-6 levels in mouse serum. The data are presented as mean ± SEM. Statistical analysis was conducted using one-way ANOVA followed by the Tukey post hoc test between three groups and using T-test between two groups. * denotes comparisons between the CON and DSS groups, ^#^ indicates comparisons between the DSS and CA groups, and ^∆^ indicates comparisons between the CON and DSS groups. * *p* < 0.05; ** *p* < 0.01; *** *p* < 0.001; **** *p* < 0.0001; ^#^ *p* < 0.05; ^##^ *p* < 0.01; ^###^ *p* < 0.001; ^####^ *p* < 0.0001; ^∆^ *p* < 0.05.

Weight loss during the modeling period is a characteristic feature indicative of colitis severity [34]. As shown in Figure 1a, mice in the DSS group experienced a continuous decline in body weight starting from the third day. In contrast, the CA group exhibited a slower rate of weight loss, which began on the fourth day. In comparison with the CON group, mice in the DSS group exhibited a significant decrease in body weight starting from the sixth day, and weight loss in the DSS group intensified with the duration of DSS administration. Interestingly, the weight of the CA group was significantly higher than that of the DSS group and CON group on the fourth day. This phenomenon may be attributed to the brief period of DSS administration before the mice started experiencing discomfort, and CA, improving the growth and development of the mice, leading to weight gain. Nevertheless, there is no other robust evidence to corroborate this, necessitating further investigation. After seven days of modeling, there was a significant decrease in the body weight of mice in the DSS group compared to the CON group (*p* = 0.0009). However, CA supplementation significantly mitigated the DSS-induced weight loss (*p* = 0.0171) (Figure 1b). Furthermore, visible bleeding in the stool was observed in the DSS group starting on the fifth day of modeling, while it occurred later in the CA group. By the seventh day post modeling, the severity of hematochezia in the CA group was notably lower than that in the DSS group (Figure 1c).

The disease activity index (DAI) was employed to assess the severity of colitis in mice, taking into account factors such as weight loss, stool consistency, and intestinal bleeding throughout the modeling period. As depicted in Figure 1d, the DAI score showed a progressive increase with the duration of DSS administration. At the conclusion of the experiment, the DAI score in the DSS group had significantly escalated, reaching a substantial value of 8.375, which was markedly higher than that in the CON group (*p* < 0.0001). Following CA treatment, the DAI score in the CA group was significantly lower than that in the DSS group (*p* = 0.0369) (Figure 1e). These observed phenotypic changes collectively indicate that CA effectively mitigates DSS-induced colitis.

### 2.2. Supplementation with CA Attenuated DSS-Induced Colon Shortening and Colonic Histological Damage

Colon length and colon histological assessment are effective disease indicators in DSS-induced colitis models. Disease severity is often correlated with a reduction in colon length due to intestinal inflammation [35]. Following seven days of DSS exposure, the colon length of mice in the DSS group was significantly shortened compared to the CON group (*p* < 0.0001), while CA supplementation significantly alleviated the DSS-induced colon length reduction (*p* = 0.0162) (Figure 1f,g). Histological examination of colon tissue stained with H&E revealed that the colon tissue in the CON group exhibited a normal histological morphology, with intact crypts and mucosal structures and no noticeable inflammatory cell infiltration. Conversely, the colonic mucosal tissue of mice in the DSS group displayed erosion and loss of epithelial integrity, accompanied by a substantial infiltration of inflammatory cells. Additionally, the crypt structure in the colon of DSS-exposed mice was disrupted. However, following CA supplementation, the mucosal structure of the colon improved, with only a minimal amount of inflammatory cell infiltration, and a reduction in crypt damage was observed (Figure 1h–j). 

The histological scoring of the colon is determined by evaluating the extent of inflammatory cell infiltration, the degree of tissue damage, and the level of crypt damage. As depicted in Figure 1k, the histopathological score of the colon in the DSS group exhibited a significant increase (*p* < 0.0001) compared to the CON group. However, the supplementation of CA significantly prevented the elevated histopathological score induced by DSS (*p* < 0.0001). In summary, these findings strongly indicate the effectiveness of CA in preventing intestinal inflammation.

### 2.3. Supplementation with CA Reduced the Levels of Inflammatory Factors in Mouse Serum

Increased intestinal permeability and the production of pro-inflammatory cytokines, which play a pivotal role in the pathogenesis of IBD, contribute to the observed inflammatory cell infiltration [1]. To further validate the beneficial effects of CA on intestinal inflammation, the levels of TNF-α and IL-6 in the serum of mice from each group were assessed. As seen in Figure 1l,m, in comparison to the CON group, the levels of TNF-α and IL-6 in the serum of the mice in the DSS group were significantly elevated (*p* < 0.0001, *p* = 0.0005). After supplementing CA, the concentrations of TNF-α and IL-6 were markedly lower than those in the DSS group (*p* = 0.0087, *p* = 0.0008). These results collectively provide additional confirmation that CA effectively mitigates DSS-induced colitis.

### 2.4. Supplementation with CA Regulated Gut Microbiota

**Figure 2:**
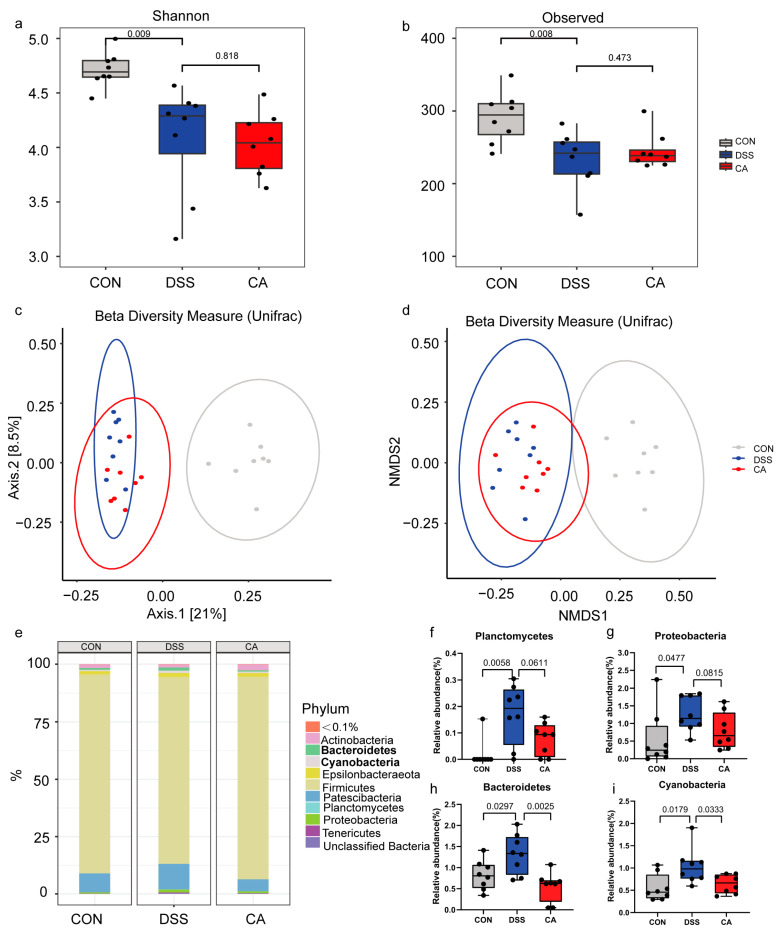

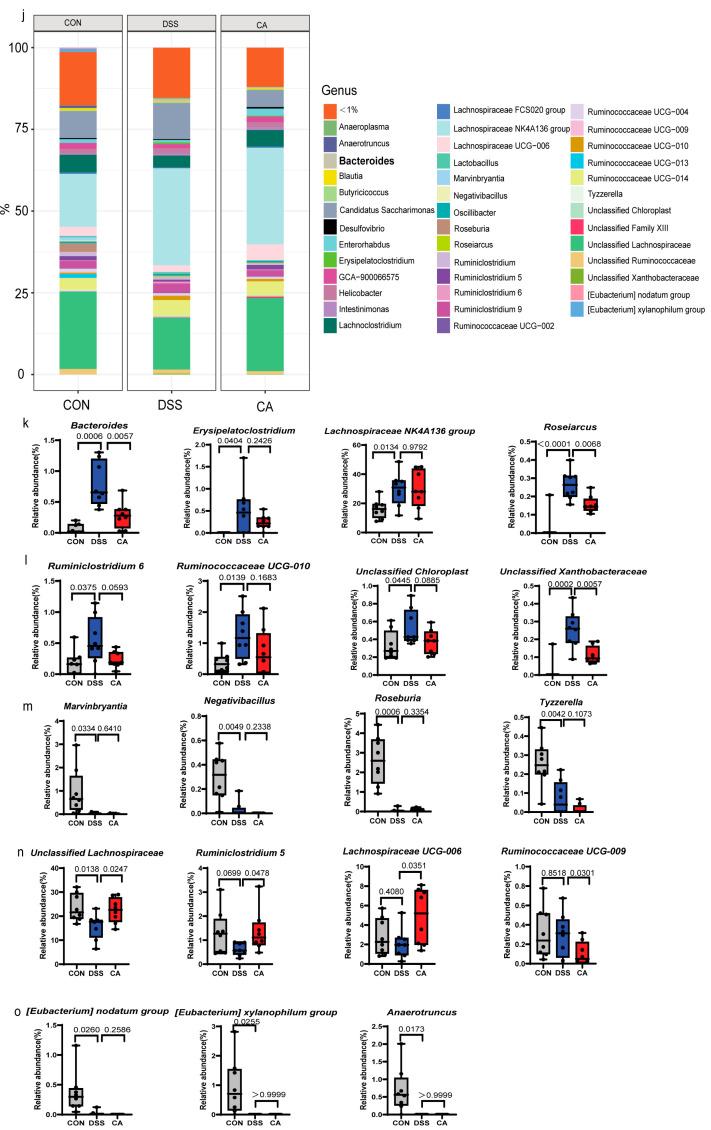
Supplementation with CA regulated gut microbiota. (**a**) Shannon index among three groups. (**b**) Observed OTUs among three groups. (**c**) Principal coordinate analysis (PCoA). (**d**) Non-metric multidimensional scaling analysis (NMDS). (**e**) Relative abundance of taxa at the phylum level. (**f**–**i**) Significant differences in the abundances of gut microbial community at the phylum level. (**j**) Relative abundance of taxa at the genus level. (**k**–**o**) Significant differences in the abundances of gut microbial community at the genus level.

The balance of gut microbiota plays a crucial role in shaping host immunity and maintaining gut homeostasis. However, when this balance is disrupted, it can lead to intestinal immune dysfunction and trigger inflammation in IBD [36]. To investigate whether CA can modulate the gut microbiota, we assessed the composition and structure of the mouse gut microbiota using 16S rRNA sequencing. The Shannon index and Observed OTUs were employed to evaluate the α-diversity of the gut microbiota. As shown in Figure 2a,b, compared to the CON group, the α-diversity of the gut microbiota was significantly reduced in the DSS group, as indicated by a decrease in the Shannon index (*p* = 0.009) and Observed OTUs (*p* = 0.008). To explore differences in the composition of the microbiota structure among samples, principal coordinate analysis (PCoA) and non-metric multidimensional scaling analysis (NMDS) were utilized to assess the β-diversity of the gut microbiota. The β-diversity analysis revealed a clear separation between the gut microbiota of the DSS and CON groups, indicating an overall change in the gut microbiota structure following DSS exposure. However, CA intervention did not alter the overall structure of the gut microbiota affected by DSS exposure (Figure 2c,d).

Subsequently, we conducted an analysis of species composition and displayed the relative abundance of different bacteria in the three groups. At the phylum level, Firmicutes and Patescibacteria were the dominant bacteria (Figure 2e). Compared to the CON group, the DSS group exhibited a significant increase in the abundance of Planctomycetes (*p* = 0.0058) and Proteobacteria (*p* = 0.0477). Although there was no significant difference in the abundance of Planctomycetes and Proteobacteria between the CA group and the DSS group, there was a noticeable downward trend (*p* = 0.0611, *p* = 0.0815). Additionally, our results showed significant differences in Bacteroidetes and Cyanobacteria among the three groups. After DSS exposure, the abundance of Bacteroidetes (*p* = 0.0297) and Cyanobacteria (*p* = 0.0179) significantly increased compared to the CON group, while CA supplementation led to a significant decrease in the abundance of Bacteroidetes (*p* = 0.0025) and Cyanobacteria (*p* = 0.0333) (Figure 2f–i). At the genus level, the dominant gut microbiota included *Candidatus Saccharimonas*, *Lachnospiraceae NK4A136 group*, and unclassified *Lachnospiraceae* (Figure 2j). Figure 2k–o illustrate significant differences in gut microbiota at the genus level among the three groups. Specifically, in the DSS group, there was a notable increase in the abundance of *Bacteroides*, *Roseiarcus*, and unclassified *Xanthobacteraceae*, while the abundance of unclassified *Lachnospiraceae* decreased significantly when compared to the CON group. On the contrary, CA supplementation led to a significant increase in the abundance of unclassified *Lachnospiraceae*, whereas the abundances of *Bacteroides*, *Roseiarcus*, and unclassified *Xanthobacteraceae* were significantly reduced in the CA group. Results from the linear discriminant analysis effect size (LEfSe) revealed distinct species among the three groups. Genus-level discriminant species in the CON group included *Roseburia*, *Ruminococcaceae UCG-013*, *[Eubacterium] xylanophilum group*, *Marvinbryantia*, *Anaerotruncus*, *[Eubacterium] nodatum group*, *Negativibacillus*, and *Candidatus Arthromitus*. In the DSS group, discriminant species included *Bacteroides*, *Erysipelatoclostridium*, and *Roseiarcus*, whereas in the CA group, discriminant species comprised *[Ruminococcus] torques group*, *Ruminococcaceae UCG-014*, and *Family XIII AD3011 group* (Figure S1). Overall, the results indicated that DSS exposure disrupted the balance of gut microbiota in mice, and CA intervention had a beneficial effect on restoring gut microbiota homeostasis.

### 2.5. Supplementation with CA Altered Fecal Metabolites in Colitis Mice

**Figure 3:**
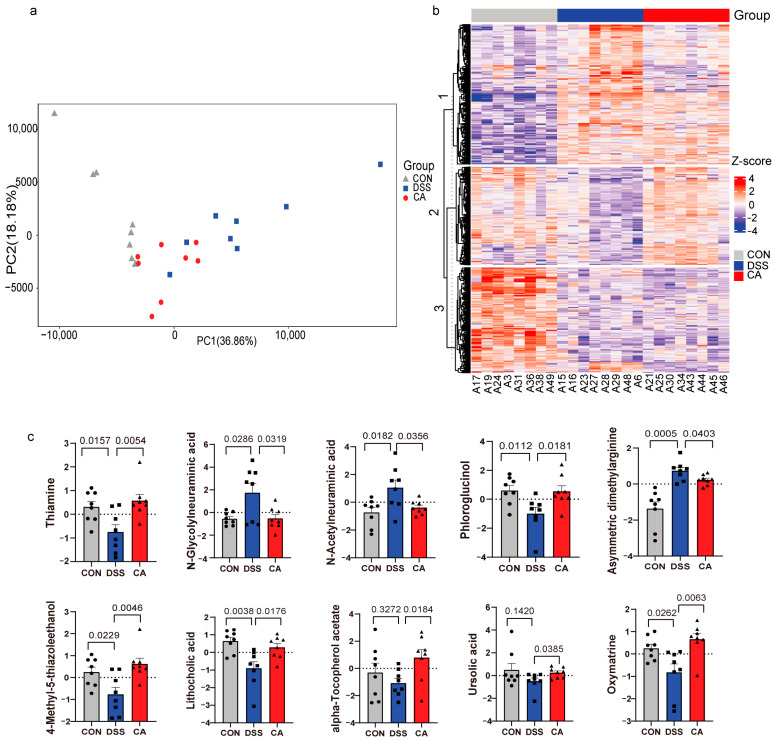
Supplementation with CA altered fecal metabolites. (**a**) Principal component analysis (PCA) score plot of metabolomic features between three groups. (**b**) Cluster analysis of differential metabolite expression level between three groups. (**c**) The metabolites with significant difference between three groups.

Since 16S rRNA sequencing revealed significant changes in certain intestinal microbiota following CA supplementation, non-targeted liquid chromatograph mass spectrometry (LC-MS) was employed to further assess potential perturbations in the fecal metabolome after CA supplementation. To assess differences between groups (the dissimilarity among samples from the three groups) and within groups (the clustering degree of samples within each group), principal component analysis (PCA) was conducted. PCA results indicated that the first two principal component scores, PC1 and PC2, accounted for 36.86% and 18.18% of the variance, respectively (Figure 3a). There was a clear separation observed among the CON group, DSS group, and CA group, suggesting that DSS treatment induced alterations in mouse fecal metabolites, while CA intervention partially mitigated these changes. Differential metabolites were identified based on three criteria: VIP ≥ 1, Fold Change ≥ 1.2 or ≤0.83, and *p*-value < 0.05. In comparison to the CON group, the DSS group exhibited 359 up-regulated and 323 down-regulated differential metabolites, totaling 682 differential metabolites between the two groups. Conversely, the CA group, when compared to the DSS group, displayed 330 up-regulated and 340 down-regulated differential metabolites, amounting to 670 differential metabolites between these two groups. Upon clustering and analyzing the expression levels of these differential metabolites, distinct expression patterns of metabolites in the three sample groups became apparent (Figure 3b). Potential biomarker metabolites were defined as differential metabolites exhibiting an opposite trend following DSS exposure and CA supplementation. Table 1 lists potential biomarker metabolites in feces following DSS exposure and CA consumption. Asymmetric dimethylarginine, N-glycolylneuraminic acid, and N-acetylneuraminic acid showed significant increases in the DSS group compared to the CON group but were significantly down-regulated after CA supplementation. Additionally, following DSS exposure, metabolites such as phloroglucinol, thiamine, 4-methyl-5-thiazoleethanol, lithocholic acid, and oxymatrine were significantly down-regulated, with CA intervention leading to significant increases in these metabolites as well. While alpha-tocopherol, acetate, and ursolic acid did not exhibit significant differences between the CON group and DSS group, a decreasing trend was observed in the DSS group compared to the CON group. However, after CA supplementation, the levels of these metabolites were significantly increased (Figure 3c). Figure S2a showed key metabolites identified by LEfSe. To elucidate the metabolic pathways in which CA participates, we conducted enrichment analyses of altered metabolites using the KEGG database. Several significant metabolic pathways were identified (Figure S2b), including “Glycine, serine and threonine metabolism”, “Arginine and proline metabolism”, “Pyrimidine metabolism”, “Tryptophan metabolism”, and “Amino sugar and nucleotide sugar metabolism”.

### 2.6. Correlation Analysis between Gut Microbiota, Fecal Differential Metabolites, and Host Phenotypes

**Figure 4:**
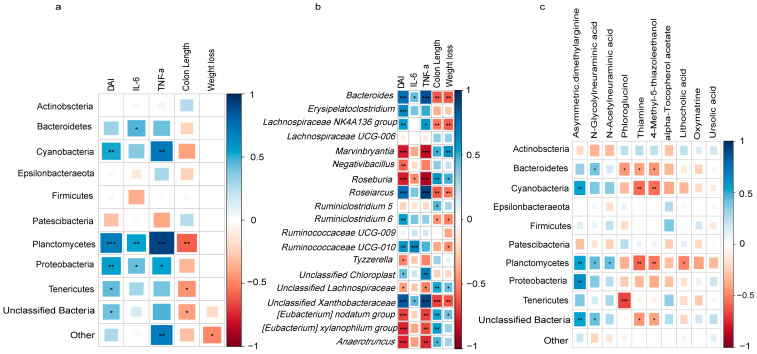
The heatmap of Spearman’s correlation analysis between three groups. (**a**) Correlation among gut microbiota and host phenotypes at the phylum level. (**b**) Correlation among gut microbiota and host phenotypes at the genus level. (**c**) Correlation among candidate metabolites and gut microbiota. * *p* < 0.05; ** *p* < 0.01; *** *p* < 0.001.

Based on the aforementioned findings, it is evident that both DSS exposure and CA intervention induced changes in the abundance of certain intestinal microbiota species and the composition of fecal metabolites in mice. To explore the interplay between the gut microbiota, host metabolism, and different treatments, Spearman correlation analysis was performed on fecal metabolites, gut microbiota, and host phenotypic characteristics. The results unveiled significant correlations between the gut microbiota and host phenotypes. At the phylum level, Cyanobacteria, Planctomycetes, and Proteobacteria exhibited positive correlations with DAI and TNF-α levels. Tenericutes and unclassified bacteria were positively correlated with DAI. Bacteroidetes, Planctomycetes, and Proteobacteria demonstrated positive correlations with IL-6 levels. Planctomycetes, Tenericutes, and unclassified bacteria displayed negative correlations with colon length. Patescibacteria were positively associated with weight loss, while Tenericutes were negatively associated with weight loss (Figure 4a). At the genus level, *Bacteroides* and unclassified *Xanthobacteraceae* showed positive correlations with DAI, IL-6, and TNF-α levels, but exhibited negative correlations with colon length and weight loss. *Marvinbryantia*, *Roseburia*, *[Eubacterium] nodatum group*, *[Eubacterium] xylanophilum group*, *Anaerotruncus*, and unclassified *Lachnospiraceae* were negatively correlated with DAI and IL-6, while they were positively correlated with colon length. *Lachnospiraceae NK4A136 group*, *Roseiarcus*, and unclassified *Chloroplast* demonstrated positive correlations with DAI and TNF-α. *Erysipelatoclostridium*, *Rumiclostridium 6*, and *Ruminococcaceae UCG-010* were positively correlated with DAI, whereas *Tyzzerella* exhibited a negative correlation with DAI. *Lachnospiraceae NK4A136 group*, *Roseiarcus*, and *Rumiclostridium 6* were inversely associated with colon length and weight loss. *Marvinbryantia*, *Roseburia*, unclassified *Lachnospiraceae*, and *[Eubacterium] nodatum group* were positively correlated with weight loss, while *Ruminococcaceae UCG-010* displayed a positive correlation with IL-6 but a negative correlation with weight loss (Figure 4b). Importantly, the results also indicated that changes in fecal metabolites induced by DSS exposure and CA intervention are closely linked to gut microbiota. Cyanobacteria, Planctomycetes, Proteobacteria, and unclassified bacteria showed positive correlations with asymmetric dimethylarginine, while Bacteroidetes, Planctomycetes, and unclassified bacteria exhibited positive correlations with N-glycolylneuraminic acid. Bacteroidetes, Cyanobacteria, Planctomycetes, and unclassified bacteria displayed negative correlations with thiamine and 4-methyl-5-thiazoleethanol. In addition, Planctomycetes exhibited a positive correlation with N-acetylneuraminic acid and a negative correlation with lithocholic acid. Both Bacteroidetes and Tenericutes showed negative correlations with phloroglucinol (Figure 4c).

## 3. Discussion

In our study, CA treatment demonstrated significant benefits in mitigating the effects of DSS-induced colitis. It effectively prevented weight loss and colon shortening, and it reduced the disease activity index (DAI) and histopathological scores that had been elevated due to DSS treatment. Additionally, CA supplementation led to the suppression of pro-inflammatory factors such as TNF-α and IL-6. Taken together, these findings provide compelling evidence of CA’s effectiveness in ameliorating UC. Furthermore, our research results strongly suggest that CA exerts its anti-colitis effects through the regulation of gut microbiota and metabolites.

CA has garnered considerable attention for its well-documented anti-inflammatory properties. It has been reported that CA mitigated LPS-induced neuroinflammation, memory impairment, and amyloid production via inhibition of the NF-κB transcription pathway [37]. Landmann et al. reported that CA pretreatment significantly inhibited the mRNA expression of inducible nitric oxidesynthase (iNOS) and TNF-α in LPS-treated RAW264.7 macrophages. In the model of acute alcohol-induced hepatic steatosis, CA has demonstrated its ability to alleviate the condition in female C57BL/6J mice. This effect is achieved through the inhibition of oxidative stress and the exertion of anti-inflammatory effects [38]. *Echinacea purpurea* L. CA extract significantly reduced collagen-induced paw swelling in rats, which exerted anti-arthritic effects by reducing the levels of TNF-α, IL-1β, and PGE-2 in rat serum, and by reducing the levels of NF-κB, TNF-α, and PGE-2 in synovium tissues of the ankle joint [33]. In line with previous findings [33,37,38], our current research results demonstrate that CA treatment effectively improves colitis, reaffirming its well-established anti-inflammatory properties.

It is widely recognized that the gut harbors a vast number of bacteria that play pivotal roles in nutrition, energy metabolism, host defense, and immune system development [39]. Normally, a greater richness and diversity in the microbiota throughout life render the organism more resilient to external environmental challenges [40]. Indeed, alterations in the quantity and quality of the gut microbiota can result in dysbiosis, leading to intestinal inflammation [40]. Our results demonstrate that the mouse gut microbiota is altered following DSS exposure, revealing a reduction in diversity, which is consistent with previous reports of reduced gut microbiota diversity in IBD patients [41]. However, CA supplementation did not reverse this reduction in diversity, suggesting that CA may not exert its anti-colitis effect by increasing the overall diversity of the gut microbiota. In the current study, at the phylum level, the DSS group exhibited a significant increase in the abundance of Planctomycetes and Proteobacteria compared to the control group. The proliferation of Proteobacteria in the intestine signifies dysbiosis or an unstable microbial community structure and contributes to intestinal inflammation. Moreover, susceptibility to colitis positively correlates with the relative abundance of intestinal Proteobacteria [42]. Importantly, a study collecting biopsy samples from six UC patients through colonoscopy and utilizing Illumina high-throughput sequencing analysis has demonstrated that Proteobacteria significantly increases in UC patients during the severe inflammation stage compared to mild or moderate inflammation stages [43]. In the 1-methyl-4-phenyl-1,2,3,6-tetrahydropyridine-induced Parkinson’s disease mouse model, CA significantly reduced the abundance of phylum Bacteroidota and genus *Parabacteroides*, as well as increased the abundance of phylum Firmicutes and genera *Lactobacillus* and *Ruminiclostridium* [44]. In a report exploring the effects of CA on non-alcoholic fatty liver disease (NAFLD) in C57BL/6 mice, CA was found to reduce Firmicutes levels and increase Bacteroidetes at the phylum level, while at the genus level, the levels of *Lactobacillus*, *Turicibacter*, and *Candidatus Saccharimonas* were reduced and the levels of *Ruminococcaceae UCG-014* and *Alloprevotella* were increased after CA treatment [45]. In our study, CA supplementation reduced the abundance of Bacteroidetes, which is consistent with previous reports. Interestingly, while CA has been shown in previous studies to have various effects on gut microbiota, our results indicate that CA supplementation reduced the abundance of Cyanobacteria at the phylum level and decreased the abundance of specific genera, such as *Bacteroides*, *Roseiarcus*, unclassified *Xanthobacteraceae*, and *Ruminococcaceae UCG-009*, while it increased the abundance of *Ruminiclostridium 5*, *Lachnospiraceae UCG-006*, and unclassified *Lachnospiraceae* at the genus level. These findings highlight the complex and context-dependent effects of CA on gut microbiota and suggest a unique impact of CA in the context of colitis. The observed inconsistencies in the effects of CA on gut microbiota could indeed be attributed to various factors, including differences in animal models, CA treatment concentrations, and treatment durations. It is reported that Cyanobacteria have been associated with potential neurotoxic or pro-inflammatory activity. A randomized controlled trial analyzed the fecal microbial profile of 50 amyotrophic lateral sclerosis (ALS) patients treated with probiotics and 50 healthy individuals treated with a placebo for 6 months. This study revealed a higher abundance of Cyanobacteria in ALS patients compared to the healthy individuals [46]. Furthermore, an analysis of microbial composition in the colorectal mucosa of 20 healthy volunteers and 31 patients with colorectal adenomatous polyps revealed a significantly higher abundance of Cyanobacteria in patients with adenomas [47]. Moreover, a clinical study collected fecal specimens from 18 infants with rotavirus (G9P8)-induced acute gastroenteritis (mean age 11.8 months) and 24 infants with human norovirus infections (GII)-induced acute gastroenteritis (mean age 8.8 months). This study demonstrated a higher abundance of Cyanobacteria in infants with viral diarrhea compared to healthy individuals [48]. In fecal samples from 38 children aged 7–17 years with pediatric Roman type III IBD who had previously completed a double-blind, randomized, placebo-controlled crossover trial (fructose versus maltodextrin), an elevated abundance of Cyanobacteria was observed after fructan ingestion compared to fructan-tolerant children [49]. These studies suggest a potential involvement of Cyanobacteria in the development of gastrointestinal and inflammatory diseases, though further research is needed to determine whether intestinal Cyanobacteria can cause diseases or whether changes in their abundance are a consequence of disease. In vivo studies have demonstrated a decrease in the abundance of *Rumiclostridium 5* in the feces of rats with necrotizing pancreatitis [50]. In a study that enrolled 13 patients with multiple kidney stones and 13 matched healthy controls, a diminished abundance of *Rumiclostridium 5* was observed in patients with kidney stones [51]. These studies suggest that a decreased abundance of *Rumiclostridium 5* could potentially contribute to the development of certain diseases. Overall, our current findings suggest that CA supplementation is beneficial in promoting the health of the gut microbial ecology to some extent.

Mouse feces encompass a diverse array of metabolites that can potentially serve as reflections of both the gut microbiota composition and the outcomes of nutrient uptake, digestion, and absorption within the gastrointestinal tract. The analysis of these fecal metabolites offers a window into the repercussions of interactions between the host and gut microbiota [52]. In our current investigation, we observed a noteworthy alteration in the fecal metabolic profile of mice following exposure to DSS and subsequent CA intervention. Our findings revealed a significant increase in the levels of asymmetric dimethylarginine, N-glycolneuraminic acid, and N-acetylneuraminic acid within the DSS-exposed group. These metabolites exhibited a significant down-regulation following CA supplementation. Notably, asymmetric dimethylarginine is a prominent inhibitor of endogenous nitric oxide synthase. Elevated levels of asymmetric dimethylarginine have been associated with oxidative stress and endothelial dysfunction [53,54]. Furthermore, a study gathered serum samples from 63 patients with colon cancer and 61 healthy individuals at the Fudan University Cancer Center. This study revealed heightened serum levels of asymmetric dimethylarginine in individuals with colon cancer [55]. Similarly, our study detected an elevation in asymmetric dimethylarginine levels in the feces of mice with colitis. On the other hand, N-glycolylneuraminic acid is a non-human glycan, and several studies have reported that the ingestion of N-glycolylneuraminic acid from red meat can trigger a heterophil-antibody-mediated inflammatory response, thus fostering the development of colitis and potentially colorectal cancer [56]. Furthermore, prior in vitro research has uncovered that N-glycolylneuraminic acid can promote the proliferation of colorectal cancer cells [56]. N-Acetylneuraminic acid, another non-human sialic acid, has been documented to induce cancer and inflammation [57].

Following exposure to DSS, we observed significant up-regulation of phloroglucinol, thiamine, 4-methyl-5-thiazoleethanol, lithocholic acid, and oxymatrine. However, upon CA intervention, these metabolites exhibited significant reductions. Phloroglucinol, categorized under phenols and derivatives, has demonstrated the ability to inhibit oxidative stress and decrease the expression levels of TNF-α, IL-1β, IL-6, and PGE2 in lipopolysaccharide-stimulated RAW264.7 cells [58]. In general, nutritional deficiencies in individuals with IBD stem from factors such as decreased appetite, malabsorption, and systemic inflammation triggered by active disease [59]. Notably, thiamine deficiency is relatively prevalent in individuals with IBD and can lead to symptoms like nausea, vomiting, and abdominal pain [60]. Furthermore, 4-methyl-5-thiazoleethanol, a natural sulfur-containing flavor compound, is a degradation product of thiamine [61]. In our study, we observed a similar pattern of change for 4-methyl-5-thiazoleethanol and thiamine. It is worth noting that lithocholic acid is a secondary bile acid produced only by microbiota, such as *Lachnospiraceae* and *Ruminococcaceae families*, which undergo a 7α-dehydroxylation reaction to generate lithocholic acid. These bile acids are synthesized in the colon and subsequently transported into the bloodstream [62,63,64]. More importantly, lithocholic acid, as a metabolite of cholesterol conversion, has been shown to promote the regeneration of the intestinal epithelium via activation of the Takeda G protein-coupled receptor 5 (TGR5) in intestinal stem cells to combat epithelial damage and subsequent barrier disruption [65]. Oxymatrine, classified as an alkaloid, has exhibited anti-inflammatory properties both in vitro and in vivo [66]. Additionally, it has been reported that oxymatrine ameliorated weight loss and histological damage induced by DSS in C57BL/6 wild-type mice [67].

Moreover, our findings revealed notable correlations between intestinal bacterial strains and fecal metabolites. Specifically, Bacteroidetes exhibited a significant positive correlation with N-glycolylneuraminic acid and a significant negative correlation with thiamine, 4-methyl-5-thiazoleethanol, and phloroglucinol. Cyanobacteria demonstrated a significant positive correlation with asymmetric dimethylarginine and a significant negative correlation with thiamine and 4-methyl-5-thiazoleethanol. Furthermore, Proteobacteria displayed a significant positive correlation with asymmetric dimethylarginine. Altogether, these comprehensive results collectively suggest that CA exerts its therapeutic effect in alleviating colitis by modulating the levels of key metabolites. CA appears to down-regulate the levels of asymmetric dimethylarginine, N-glycolylneuraminic acid, and N-acetylneuraminic acid, while simultaneously up-regulating the levels of phloroglucinol, thiamine, 4-methyl-5-thiazoleethanol, lithocholic acid, and oxymatrine.

Nevertheless, this study has certain limitations. While animal models can replicate human diseases, disparities in physiology and immune systems between animals and humans may exist. Therefore, crucial confirmation of the therapeutic efficacy of CA in human colitis necessitates future clinical trials. While we observed a correlation between microbiota and metabolites in mice, the causal relationship between these factors remains unclear. Therefore, further investigations are warranted to elucidate the intricate relationship between colonic microorganisms and metabolites. Such elucidation would provide deeper insights into the mechanisms underlying the anti-colitis effects of CA. Furthermore, in our experiments, DSS-induced enteritis was conducted for a duration of only one week. This limited timeframe may not comprehensively capture all stages of colitis onset and progression. Extending the experimental duration could contribute to a more thorough understanding of the disease evolution and therapeutic effects. Importantly, although CA may confer benefits in colitis, its dosage and safety require careful consideration. Varied dosages and treatment durations may yield divergent effects and potentially induce adverse reactions. Consequently, future research on the safety profile of CA is imperative.

## 4. Materials and Methods

### 4.1. Materials

Chicoric acid (≥98%) was procured from Chengdu Pufei De Biotech Co., Ltd. (Chengdu, China). Dextran sulfate sodium salt (DSS) with a molecular weight range of 36,000–50,000 was supplied by MP Biomedical (Irvine, CA, USA). Sodium carboxymethyl cellulose (CMC) was sourced from Sinopharm Chemical Reagent Co., Ltd. (Shanghai, China). The urine fecal occult blood test kit was obtained from Nanjing Jiancheng Bioengineering Institute (Nanjing, China). Enzyme-linked immunosorbent assay (ELISA) kits were purchased from Jiubang Biotechnology Co., Ltd. (Quanzhou, China).

### 4.2. Animals and Experimental Design

Seven-week-old male BALB/c mice purchased from Cavens Experimental Animal Co., Ltd. (Changzhou, China) were housed in the Experimental Animal Center of Jiangsu University under specific pathogen-free (SPF) conditions. Mice were kept in a room with a 12 h light–dark cycle at constant temperature (20–25 °C) and humidity (40–60%) with free access to water and food. After one week of adaptation, the mice were randomly assigned to three groups: control (CON) group, dextran sulfate sodium salt (DSS) treatment group, and chicoric acid (CA) treatment group. The CA group received oral administration of chicoric acid (50 mg/kg) once daily for 14 days, while the CON and DSS groups received an equivalent 0.5% CMC solution. Seven days after the initiation of oral pre-treatment, except for the CON group, which had unrestricted access to sterile water, the other two groups were given 2.5% DSS solution to induce colitis for the final 7 days of the experiment. Importantly, the DSS solution was freshly prepared every two days. Throughout this study, the mice were monitored daily, and their body weights were recorded. All animal experiments in this study were conducted in accordance with the Guide for the Care and Use of Laboratory Animals of Jiangsu University.

After the experiment, the mice were euthanized following standard protocols. Serum, colon tissue, and fecal samples were collected and stored in a −80 °C freezer until further analysis. A portion of the colon segment was also preserved in a 4% paraformaldehyde fixative solution for subsequent investigations.

### 4.3. Disease Activity Index (DAI) Assessment

The disease activity index (DAI) score was employed to assess the severity of colitis, primarily encompassing parameters such as weight loss, stool consistency, and intestinal bleeding. Weight loss was calculated as the percentage difference between initial body weight before modeling and body weight on any day after modeling (0: 0–1%; 1: 1–5%; 2: 5–10%; 3: 10–15%; 4: 15% or more). The consistency of stool was visually evaluated and classified based on the following criteria (0: normal feces; 1: soft but formed stool; 2: very soft and shapeless stool; 3: semi-diarrhea; 4: full diarrhea). Intestinal bleeding was detected with urine fecal occult blood test kit (0: the color of the stool does not change; 1: turn blue-green in 30 s; 2: turn blue-green in 10 s; 3: instantly turn blue-green; 4: instantly turn blue) [68]. The DAI score was calculated by summing the individual scores for these three parameters.

### 4.4. Colonic Hematoxylin and Eosin (H&E) Staining and Histopathological Analysis

The mouse colon was isolated and photographed, and its length was measured. A segment of the colon, obtained from the same region, was fixed in a 4% paraformaldehyde fixative solution. Following paraffin embedding, the colon tissue was sectioned into 5 μm-thin slices and stained with hematoxylin and eosin. Visualization was carried out using a Leica Microsystems microscope (Leica DM6000 B) for subsequent pathological analysis. Furthermore, histological score refers to the following criteria based on the severity of inflammatory cell infiltration, extent of damage, and degree of crypt damage. The severity of inflammatory cell infiltration was graded as follows: 0 for absent, 1 for slight and scattered cell infiltration, 2 for moderate cell infiltration with visible cell clusters, and 3 for extensive inflammatory cell infiltration resulting in loss of tissue structure. The extent of damage was scored as 0 for no damage, 1 for mucosal damage, 2 for mucosal and submucosal damage, and 3 for transmural damage. The degree of crypt damage was categorized as 0 for intact crypts, 1 for basal one-third damage, 2 for basal two-thirds damage, 3 for only superficial epithelial cells remaining intact, and 4 for complete loss of crypt and epithelial cells. The histological score was the sum of these three individual scores [69].

### 4.5. Inflammatory Cytokine Assay

The serum samples from the mice were retrieved from the −80 °C storage and allowed to thaw. Levels of TNF-α and IL-6 in mouse serum were assessed using an ELISA kit. In this assay, serum samples and horseradish peroxidase (HRP)-labeled detection antibodies were successively introduced into microwells that had been pre-coated with TNF-α or IL-6 capture antibodies. The mixture was incubated at 37 °C and subsequently subjected to thorough washing. Color development was initiated using the substrate 3,3′,5,5′-Tetramethylbenzidine (TMB), which, under the catalytic action of peroxidase, converted into a blue hue. Eventually, it transitioned into a final yellow color upon exposure to an acid. The absorbance at 450 nm was quantified using a microplate reader (Tecan Infinite^®^ 200 PRO) within 15 min after the addition of the stop solution. Subsequently, the concentration of each sample was determined.

### 4.6. 16S rRNA DNA Sequencing

Feces samples of mice in each group were selected to extract deoxyribonucleic acid (DNA), and qualified genomic DNA samples were taken to perform polymerase chain reaction (PCR) amplification on the V3-V4 region (338F ACTCCTACGGGAGGCAGCAG, 806R GGACTACHVGGGTWTCTAAT) of the bacteria. Additionally, the PCR amplification products were purified using Agencourt AMPure XP magnetic beads and were then eluted in Elution Buffer to prepare the library. The fragment size distribution and concentration of the libraries were assessed using an Agilent 2100 Bioanalyzer (equipped with Agilent 2100 Expert software version B.02.08) Qualified libraries, based on insert size, were subsequently subjected to sequencing on the HiSeq platform. The raw 16S rRNA sequences were imported into Quantitative Insights Into Microbial Ecology version 2 (QIIME2) software for demultiplexing and quality filtering. Divisive Amplicon Denoising Algorithm (DADA2) was employed for denoising sequences and for the construction of a feature table. A custom Naïve Bayesian classifier was developed for taxonomic classification against the SILVA database release 132, utilizing the amplified 16S region. Subsequently, sequences were clustered into Operational Taxonomic Units (OTUs) based on their similarity. A sampling depth, defined as the number of sequences in the sample with the fewest sequences, was employed to generate Observed OTUs as an α diversity measure and Sim distances as a β diversity measure. The Kruskal–Wallis test was employed to assess differences in α diversity. Differences in β diversity were assessed using permutation-based analysis of variance, followed by Benjamini–Hochberg (BH) correction. Microbiome composition data were obtained by normalizing to the total number of reads in each sample (relative abundance). Analysis of compositions of microbiomes with bias correction was utilized for the differential abundance analysis at the phylum and genus levels, with significance set at *p* < 0.05. To determine the significantly important microbial taxa, the linear discriminant analysis effect size (LEfSe) analysis was carried out as previously reported [70].

### 4.7. Untargeted Metabolomics Analysis

Twenty-five milligrams of thawed fecal samples were placed in a 1.5 mL Eppendorf tube and mixed with 800 uL of pre-cooled extracting solution (−20 °C) composed of methanol/acetonitrile/water in a 2:2:1 (*v*/*v*/*v*) ratio, along with 10 uL of an internal standard. The mixture was then ground for 5 min. Afterward, the samples were sonicated for 10 min at 4 °C and allowed to stand at 20 °C for 1 h. Subsequently, the mixtures were centrifuged at 25,000× *g* for 15 min at 4 °C. The supernatant was collected, freeze-dried, and then redissolved in 600 uL of a mixed solution (methanol/water = 1:9, *v*/*v*). Following thorough mixing, the solution was centrifuged at 25,000× *g* for 15 min at 4 °C, and the resulting supernatant was retained.

The supernatant from each sample was collected and combined to create a quality control (QC) sample. Metabolite separation and detection were performed using a Waters UPLC I-Class Plus system (Waters, Milford, MA, USA) coupled to a Q Exactive high-resolution mass spectrometer (Thermo Fisher Scientific, Waltham, MA, USA). A BEH C18 column (1.7 μm, 2.1 × 100 mm, Waters, USA) was employed in this experiment. For the positive ion mode mobile phase, liquid A consisted of an aqueous solution with 0.1% formic acid, while liquid B was composed of methanol containing 0.1% formic acid. In contrast, for the negative ion mode mobile phase, liquid A contained an aqueous solution with 10 mM ammonium formate, and liquid B consisted of a solution containing 10 mM ammonium formate in 95% methanol. Gradient elution was conducted according to the following conditions: 0 to 1 min, 2% B solution; 1 to 9 min, a gradient from 2% to 98% B solution; 9 to 12 min, 98% B solution; 12 to 12.1 min, a transition from 98% B solution to 2% B solution; and 12.1 to 15 min, 2% B solution. The flow rate was set at 0.35 mL/min, the column temperature was maintained at 45 °C, and the injection volume was 5 μL.

The acquisition of primary and secondary mass spectrometry data was conducted using a Q Exactive mass spectrometer. The primary resolution was set at 70,000, with an automatic gain control (AGC) target of 3 × 10^6^ and a maximum injection time of 100 ms. Fragmentation for secondary mass spectrometry was based on precursor ion intensity, selecting the top 3 for fragmentation. The secondary resolution differed from the primary, being set at 17,500, with an AGC target of 1 × 10^6^ and a maximum injection time of 50 ms. Fragmentation energy levels were configured at 20, 40, and 60 eV. Additionally, the sheath gas had a flow rate of 40, and the auxiliary gas was set to a flow rate of 10. In the positive ion mode, the spray voltage was 3.80 |KV|, whereas in the negative ion mode, it was 3.20 |KV|. The ion transfer tube temperature was adjusted to 320 °C, and the temperature of the auxiliary gas heating was set to 350 °C. 

The raw data were imported into Compound Discoverer 3.3 software, provided by Thermo Fisher Scientific, USA. Concurrently, mass spectrometry data analysis was carried out using the BMDB (BGI Metabolome Database), mzCloud database, and ChemSpider online database. In the R 4.0.2 software environment, the MetaX package was employed to conduct principal component analysis (PCA) and partial least squares discriminant analysis (PLS-DA) using the data derived from Compound Discoverer 3.3 software. The Variable Importance in Projection (VIP) obtained from the PLS-DA model, the Fold Change calculated as the ratio of the mean expression of each metabolite between two groups, and the P-value obtained from Student’s T-test were utilized to assess statistical significance. The criteria for identifying differential metabolites were as follows: (1) VIP ≥ 1, (2) Fold Change ≥ 1.2 or ≤0.83, (3) *p*-value < 0.05 [71,72]. Enrichment analysis of differential metabolites was conducted based on the KEGG database (https://www.metaboanalyst.ca/MetaboAnalyst/ModuleView.xhtml (accessed on 28 December, 2023)).

### 4.8. Statistical Analysis

The results are presented as mean ± SEM (standard error of the mean), and data analysis was performed using GraphPad Prism 9.0 software. Statistical comparisons among the three groups were conducted using one-way ANOVA, with a *p*-value of <0.05 considered statistically significant. Phenotypic data, 16s rRNA sequencing results, and the metabolite dataset in each group were used for correlation analysis. The Spearman correlation coefficients were calculated between the data. Spearman correlation analysis was conducted using the R 4.0.2 software with the Corrplot and Psych package.

## 5. Conclusions

To conclude, this study establishes that the targeted administration of CA can alleviate DSS-induced colitis in mice. The anti-colitis effect of CA is, at the very least, partially mediated through the modulation of gut microbiota and metabolic processes. The structure of the gut microbiota was restored after CA supplementation, including reductions in levels of Bacteroidetes and Cyanobacteria at the phylum level and *Bacteroides*, *Roseiarcus*, and unclassified *Xanthobacteraceae* at the genus level, while the abundance of unclassified *Lachnospiraceae* at the genus level was increased. In addition, the metabolome results showed that CA prevents colitis by down-regulating asymmetric dimethylarginine, N-glycolylneuraminic acid, and N-acetylneuraminic acid, and up-regulating phloroglucinol, thiamine, 4-methyl-5-thiazoleethanol, lithocholic acid, and oxymatrine. In essence, this current study provides a critical foundation for considering CA as a promising functional food ingredient for combating colitis.

## Figures and Tables

**Table 1:** Potential biomarkers after DSS exposure and CA supplementation.

Metabolite	Fold Change	*p*-Value	VIP	DSS vs. CON	CA vs. DSS	CA vs. CON
Asymmetric dimethylarginine	0.6509	0.0403	1.2106	↑	↓	↑
D-(+)-Raffinose	0.4744	0.0071	1.5651	↑	↓	↑
N-Acetyl-D-glucosamine	0.4189	0.0348	1.5544	↑	↓	n.s.
Quinone sulfate	0.1482	0.0248	3.6340	↑	↓	n.s.
Xanthurenic acid	0.245	0.0126	2.1214	↑	↓	n.s.
4-hydroxy-2-quinolinecarboxylic acid	1.8232	0.0039	1.5071	↓	↑	n.s.
1H-Indole-2,3-dione	0.5489	0.0261	1.4475	↑	↓	n.s.
Gamma-linolenic acid	0.6312	0.0408	1.1922	↑	↓	n.s.
Thiamine	2.0784	0.0054	1.4288	↓	↑	n.s.
4-Methyl-5-thiazoleethanol	2.1456	0.0046	1.5127	↓	↑	n.s.
N-Glycolylneuraminic acid	0.0199	0.0319	2.9523	↑	↓	n.s.
Allantoin	0.0886	0.0491	2.8324	↑	↓	n.s.
N-Acetylneuraminic acid	0.1702	0.0356	2.0773	↑	↓	n.s.
Phloroglucinol	3.5918	0.0181	2.0928	↓	↑	n.s.
Alpha -Aspartylphenylalanine	0.2381	0.0487	1.1902	↑	↓	n.s.
Lithocholic acid	1.9365	0.0176	1.4303	↓	↑	n.s.
Lipoic acid	0.5566	0.0216	1.2972	↑	↓	n.s.
D-(−)-Quinic acid	0.3136	0.0196	1.8506	↑	↓	n.s.
L-Alanine	0.3494	0.0040	2.0185	↑	↓	n.s.
Piperine	1.3955	0.0440	1.2827	↓	↑	n.s.
Oxymatrine	2.4038	0.0063	1.2889	↓	↑	n.s.
2-Hydroxy-4-(hydroxymethyl)-6-(1-hydroxy-3-methylbut-2-enyl)-3-[(E)-prop-1-enyl]-7-oxabicyclo[4.1.0]hept-3-en-5-one	1.8753	0.0037	1.4079	↓	↑	n.s.
Ibuprofen	0.3316	0.0118	1.4320	↑	↓	n.s.
15-Deoxy-delta 12,14-prostaglandins D2	2.74	0.0003	2.0166	↓	↑	n.s.
Thymidine 5′-monophosphate	0.1015	0.0162	2.6353	↑	↓	n.s.
Arachidonoyl ethanolamide phosphate	0.5875	0.0201	1.5616	↑	↓	n.s.
(S)-AL 8810	0.4339	0.0208	2.1068	↑	↓	n.s.
14-(Hydroxymethyl)-5,9-dimethyltetracyclo[11.2.1.0]	1.535	0.0295	1.2260	↓	↑	n.s.
11-Deoxy prostaglandin F2	0.4026	0.0184	1.2333	↑	↓	n.s.
Skatole	0.7237	0.0074	1.1165	n.s.	↓	n.s.
Alpha-tocopherol acetate	16.971	0.0184	2.6740	n.s.	↑	n.s.
Ursolic acid	1.5638	0.0385	1.1528	n.s.	↑	n.s.

The values of Fold Change, *p*-value, VIP are all from the comparison between CA group and DSS group. ↑ represents that the metabolite is up-regulated in the comparison of the two groups. ↓ represents that the metabolite is down-regulated in the comparison of the two groups. n.s. represents no significant difference between the two groups.

## Data Availability

Data are contained within the article.

## Supplementary Materials

The following supporting information can be downloaded at https://www.mdpi.com/article/10.3390/ijms25020841/s1.

## Abbreviations

CA	Chicoric acid
DSS	Dextran sulfate sodium
DAI	Disease activity index
TNF-α	Tumor necrosis factor-α
IL-6	Interleukin- 6
IBD	Inflammatory bowel disease
UC	Ulcerative colitis
CD	Crohn’s disease
SCFAs	Short-chain fatty acids
NF-κB p65	Nuclear factor kappa-B p65
NF-κB	Nuclear factor kappa-B
LRR	Leucine-rich repeat
PYD	Pyrin domain
LPS	Lipopolysaccharide
IL-1β	Interleukin -1β
PGE-2	Prostaglandin E2
CMC	Sodium carboxymethyl cellulose
ELISA	Enzyme-linked immunosorbent assay
SPF	Specific pathogen-free
CON	Control
HRP	Horseradish peroxidase
TMB	3,3′,5,5′-Tetramethylbenzidine
H&E	Colonic hematoxylin and eosin
QC	Quality control
AGC	Automatic gain control
DNA	Deoxyribonucleic acid
PCR	Polymerase chain reaction
QIIME2	Quantitative Insights Into Microbial Ecology version 2
PCoA	Principal coordinate analysis
NMDS	Non-metric multidimensional scaling analysis
DADA2	Divisive Amplicon Denoising Algorithm
LC-MS	Liquid chromatograph mass spectrometer
PCA	Principal component analysis
LEfSe	Linear discriminant analysis effective size
PLS-DA	Partial least squares discriminant analysis
VIP	Variable Importance in Projection
DA score	Differential abundance score
OTUs	Operational Taxonomic Units
iNOS	Inducible nitric oxidesynthase
ALS	Amyotrophic lateral sclerosis
NAFLD	Non-alcoholic fatty liver disease
TGR5	Takeda G protein-coupled receptor 5
