# Supplementary material for: Chicoric Acid Effectively Mitigated Dextran Sulfate Sodium (DSS)-Induced Colitis in BALB/c Mice by Modulating the Gut Microbiota and Fecal Metabolites
Source: Int J Mol Sci. 2024 Jan 10;25(2):841. doi: 10.3390/ijms25020841 (PMC10815209; doi:10.3390/ijms25020841)
Supplement: Supplementary file 1 [file ijms-25-00841-s001.zip › ijms-2796582-supplementary.pdf]

Supplementary Materials

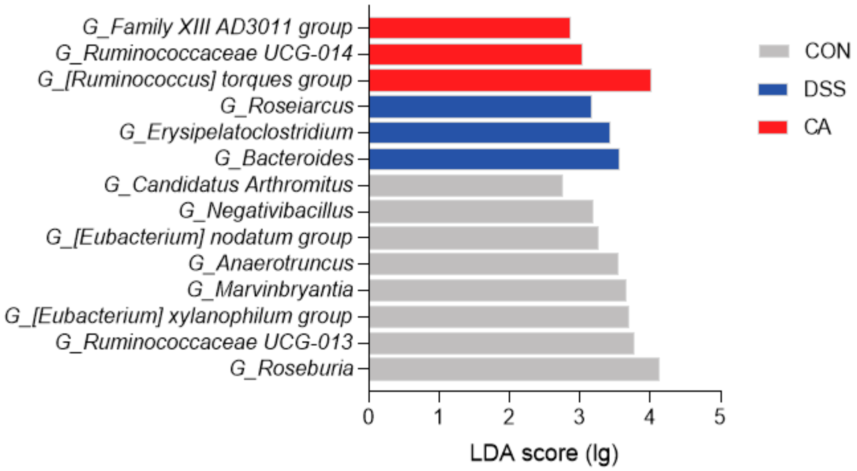

Figure S1. LEfSe analysis on genus levels. Species were displayed that exhibited significant differences among groups, characterized by LDA values greater than 2.5.

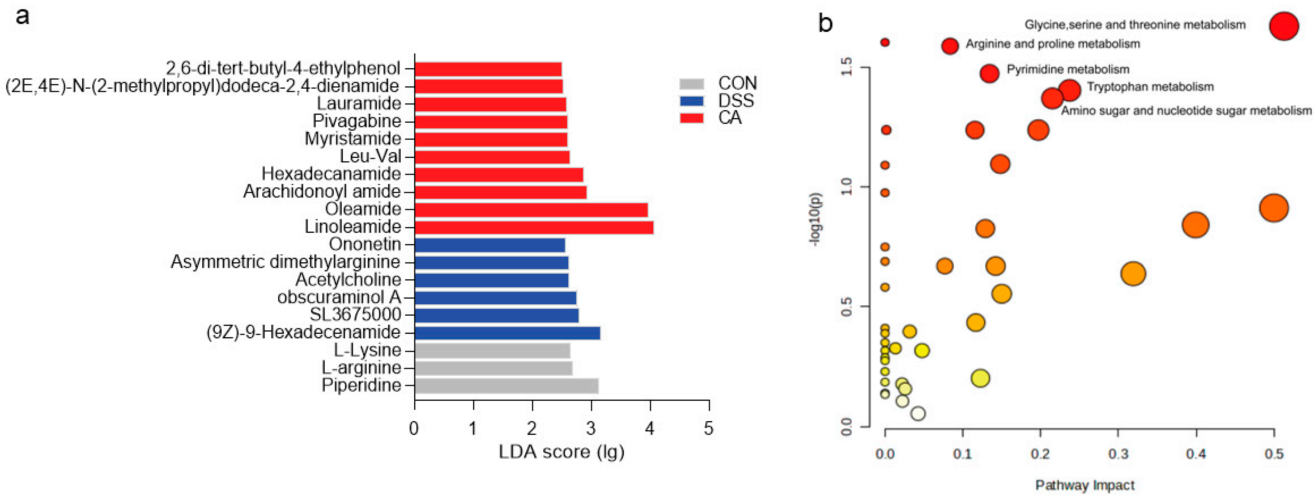

Figure S2. (**a**) Key metabolites identified by LEFse analysis. Metabolites characterized by LDA values greater than 2.5. (**b**). KEGG pathway analysis of differential metabolites in the CA vs DSS group. Each dot on the graph represents a distinct KEGG pathway, with dot size corresponding to the number of metabolites in the pathway. Larger dots indicate a higher metabolite count. The color of each dot reflects the *p*-value magnitude, with darker colors indicating smaller *p*-values.
